# Protein Profiling Gastric Cancer and Neighboring Control Tissues Using High-Content Antibody Microarrays

**DOI:** 10.3390/microarrays5030019

**Published:** 2016-07-08

**Authors:** Martin Sill, Christoph Schröder, Ying Shen, Aseel Marzoq, Radovan Komel, Jörg D. Hoheisel, Henrik Nienhüser, Thomas Schmidt, Damjana Kastelic

**Affiliations:** 1 Division of Biostatistics, Deutsches Krebsforschungszentrum (DKFZ), Im Neuenheimer Feld 581, 69120 Heidelberg, Germany; m.sill@dkfz.de; 2 Division of Functional Genome Analysis, Deutsches Krebsforschungszentrum (DKFZ), Im Neuenheimer Feld 580, 69120 Heidelberg, Germany; schroeder@sciomics.de (C.S.); a.marzoq@dkfz.de (A.M.); j.hoheisel@dkfz.de (J.D.H.); 3 Sciomics GmbH, In Neuenheimer Feld 583, 69120 Heidelberg, Germany; 4 Department of General, Visceral and Transplantation Surgery, University Clinic Heidelberg, Im Neuenheimer Feld 110, 69120 Heidelberg, Germany; shenying_0624@outlook.com (Y.S.); Henrik.Nienhueser@med.uni-heidelberg.de (H.N.); Thomas1.schmidt@med.uni-Heidelberg.de (T.S.); 5 Medical Faculty, University of Ljubljana, Vrazov Trg 2, 1000 Ljubljana, Slovenia; komel@mf.uni-lj.si

**Keywords:** gastric cancer, adenocarcinoma, affinity based proteomics, antibody microarrays, biomarker identification

## Abstract

In this study, protein profiling was performed on gastric cancer tissue samples in order to identify proteins that could be utilized for an effective diagnosis of this highly heterogeneous disease and as targets for therapeutic approaches. To this end, 16 pairs of postoperative gastric adenocarcinomas and adjacent non-cancerous control tissues were analyzed on microarrays that contain 813 antibodies targeting 724 proteins. Only 17 proteins were found to be differentially regulated, with much fewer molecules than the numbers usually identified in studies comparing tumor to healthy control tissues. Insulin-like growth factor-binding protein 7 (IGFBP7), S100 calcium binding protein A9 (S100A9), interleukin-10 (IL‐10) and mucin 6 (MUC6) exhibited the most profound variations. For an evaluation of the proteins’ capacity for discriminating gastric cancer, a Receiver Operating Characteristic curve analysis was performed, yielding an accuracy (area under the curve) value of 89.2% for distinguishing tumor from non-tumorous tissue. For confirmation, immunohistological analyses were done on tissue slices prepared from another cohort of patients with gastric cancer. The utility of the 17 marker proteins, and particularly the four molecules with the highest specificity for gastric adenocarcinoma, is discussed for them to act as candidates for diagnosis, even in serum, and targets for therapeutic approaches.

## 1. Introduction

Gastric cancer is currently the second leading cause of cancer-related death worldwide [1]. The median survival time is 24 months and the overall five-year survival rate is between 10% and 30%. Mortality has remained unchanged over the past three decades. Curative therapy commonly involves surgical resection of the complete stomach or part of it. However, the response is quite poor [2]. This can be attributed to an extended asymptomatic period and the resulting difficulty in detecting early stages of the disease. Early diagnosis improves the long-term survival of patients substantially. Also, a single therapeutic target—human epidermal growth factor receptor 2 (HER2)—is currently used for gastric cancer treatment [3]. The identification of potential target molecules of high specificity is therefore urgently required.

Gastric cancer is a heterogeneous disease, a fact that is reflected by its clinical and pathological classification. The heterogeneity is responsible for an individually widely diverse clinical outcome [4]. The vast majority (95%) of gastric cancer cases are adenocarcinomas [5]. They arise from secretory epithelia in the inner lining of gastric mucosa, which can be subdivided into intestinal and diffuse (as well as indeterminate) types according to the Lauren classification [6]. Based on tumor invasiveness and differentiation, the adenocarcinomas are graded into four subtypes, T1 to T4. An alternative classification system of the World Health Organization divides gastric cancer into papillary, tubular, mucinous and poorly cohesive (as well as mixed) carcinomas [7]. Most of the adenocarcinomas diagnosed belong to the advanced cancer stages T3 and T4 and are accompanied by lymph node metastases. Without tumor resection, they exhibit a median survival period of merely five months [8].

Currently, gastric adenocarcinomas are often diagnosed on the basis of the localization of the tumor cells and their invasiveness. There are very few molecular biomarkers available to grade gastric cancer according to its invasiveness [8]. Growth factor receptors such as EGFR, c-met and ERBB2 have been used as predictors of later stages of malignancy, but with a high degree of uncertainty. CCL18 combined with particular T-cell receptors is employed as a prognostic indicator in early stages of gastric cancer [9]. A recent study performed by The Cancer Genome Atlas Research Network (TCGA) [10] revealed four classes of gastric cancer based on a combined analysis of somatic copy number, whole genome sequencing, DNA methylation profiling, mRNA and miRNA sequencing as well as results from reverse phase protein arrays. A new molecular classification was suggested, dividing gastric cancer into four subtypes: (i) tumors positive for Epstein-Barr virus, which display recurrent *PIK3CA* mutations; (ii) extreme DNA hypermethylation and amplification of *JAK2*, *CD274* and *PDCD1LG2*; (iii) microsatellite unstable tumors, which show elevated mutation rates, including mutations of genes encoding targetable oncogenic signaling proteins; and (iv) tumors with chromosomal instability, which show marked aneuploidy and focal amplification of receptor tyrosine kinases. This classification scheme underlines the complexity of gastric cancer molecular fingerprints. The high degree of gastric cancer heterogeneity emphasizes the urgent need for robust molecular classifiers as well as new potential targets for therapy, which are common between tumors and exhibit a high degree of specificity for cancer.

As indicated by the TCGA study, omics-technologies can act as excellent tools for the identification of biomarkers. DNA microarrays and, more recently, sequencing were widely employed for screening studies on biomarkers at genomic and transcript levels. However, inconsistencies in the validation of biomarkers identified by various genomic approaches have been reported. This fact as well as substantial advances in technology led recently to the strong emergence of proteome analyses for biomarker discovery. Rather than analyzing the basic information encoded in the DNA sequence, proteome analyses study molecular end products that are actually functional and thus responsible for many vital cellular activities. While the basic molecular reasons for the occurrence of tumors may vary widely, particular functional aspects are likely to be common nevertheless. Also for this reason, proteins are the molecule class that is targeted by most cancer drugs.

Affinity-based analysis processes are widely used in studies that aim at the discovery of protein biomarkers, irrespective of the actual assay format. Mass spectrometry or Western blotting, for example, take advantage of antibodies for purification or detection, respectively. Antibody microarrays are the most complex platform for affinity-based protein analysis. During the last 15 years, they have developed enormously by optimization of many experimental factors and facets—from efficient antibody attachment and blocking procedures, via improved sample isolation and labeling protocols, to incubation and detection processes that allow quantitative measurements. Meanwhile, they permit a highly sensitive, accurate and reproducible detection in a highly multiplexed manner of proteins isolated from any type of specimen, even if available sample quantities are very small [11,12,13]. The number of targets that have been studied in an individual analysis varies from several dozens to thousands [14]. Technically, there is basically no upper limit in the number of molecules that can be dealt with in parallel. The current most limiting factor is the availability of appropriate antibodies, although there are some 2.37 million binders listed in the database antibodypedia (version 9 of 23 March 2016; www.antibodypedia.com). With respect to quality assessment, elaborate procedures have been established which permit antibody microarray analyses to be performed with an accuracy and reproducibility that meet the standards for application in cancer diagnosis and prognosis in a clinical setting (e.g., [15,16]). Read-out sensitivities down to attomolar concentration or even single-molecule detection have been reported [17,18].

As an immunoassay, antibody microarrays are close to assay formats that are well established and accepted in clinical diagnostics. Thus, they represent an effective tool for biomarker discovery and also warrant a relatively quick and simple translation of results into a format that is applicable in clinical practice. We have used high-content antibody microarrays to profile gastric tumor and adjacent non-tumorous tissue samples. In most studies, the control samples are isolated from another group of tissue donors, thus adding another degree of complexity. The approach taken here should allow the identification of biomarkers of the highest possible specificity in tumor tissue; ignoring personal differences, for example, such molecules could be most useful for common diagnostic and therapeutic purposes. Immunohistochemistry was then applied to confirm the variations in the abundance of proteins, which were found to be the most discriminative.

## 2. Materials and Methods

### 2.1. Protein Isolation and Labeling

Sixteen primary tumors from patients diagnosed with gastric adenocarcinoma, who had not received any treatment prior to tumor resection, were obtained at the Department of Gastroenterology of the University Medical Centre in Ljubljana. During the resection process, also adjacent tissue surrounding each tumor was removed. Written informed consent had been given by the patients and sample collection had been approved by the institutional ethics committee. The tissue pairs were immediately snap-frozen in liquid nitrogen and stored at −80 °C until use. Total protein extract was prepared and fluorescently labeled as described in detail previously [19]. Briefly, the tissues were pulverized while still frozen in the presence of liquid nitrogen. The material was lysed for 30 min on ice in 10 volumes of lysis buffer composed of 50 mM carbonate buffer (pH 8.5), 20% glycerol, 1.0 mM MgCl_2_, 5.0 mM EDTA, 1.0 mM phenylmethanesulfonyl fluoride, 1.0 U/mL benzonase (Merck Biosciences, Schwalbach, Germany), Halt protease and phosphatase inhibitor mixture (Thermo Scientific, Bonn, Germany), 0.5% Nonidet P-40 substitute, 1.0% cholic acid, 0.25% n-dodecyl-maltoside (GenaXXon Bioscience, Ulm, Germany), and 0.5% amidosulfobetaine-14. Cell debris was pelleted by 20 min centrifugation at 4 °C and 13,000 rpm. The protein concentration was determined with the Bicinchoninic Acid Protein Assay Reagent (BCA) kit (Thermo Scientific) according to the manufacturer’s protocol. Before labeling, each protein extract was adjusted to a concentration of 1 mg/mL.

Each sample of extracted proteins was separately fluorescently labeled by reaction with the NHS-esters of the dyes DY-549 and DY-649 (Dyomics, Dresden, Germany) at a molar dye/protein ratio of 1:18, assuming that the average weight of a protein is 60 kDa. Labeling was carried out in lysis buffer in the dark at 4 °C for 1 h. Then, unreacted dye was quenched by adding 2.5% hydroxylamine for 30 min at 4 °C. The non-incorporated and inactivated dye molecules were removed with a zeba spin-desalting column with a cut-off of 3.5 kDa (Thermo Scientific). The labeled protein samples were stored frozen at −20 °C in the presence of protease inhibitor cocktail (Roche Diagnostics, Mannheim, Germany). To exclude any bias potentially introduced by the fluorescent dyes, the labels were swapped between tumor and control samples.

### 2.2. Microarray Production

The antibody microarray used for this study contained 813 antibodies directed against 724 cancer-related proteins [15]. The antibodies had been selected based on previous transcription profiles obtained from different cancer entities as well as a literature and database search on cancer-related proteins. The antibodies (1 mg/mL) were printed in quadruplicates on epoxysilane-coated slides (Nexterion-E, Schott, Jena, Germany) using the contact printer (MicroGrid-2, Biorobotics, Cambridge, UK) with SMP3B pins (Telechem, Sunnyvale, CA, USA) at a humidity of 40% to 45% in 50 mM carbonate buffer (pH 8.5), containing 0.005% Tween-20, 5% trehalose and 1 mM magnesium chloride. After spotting, the slides were allowed to equilibrate at a humidity of 40% to 45% overnight and then stored at 4 °C in dry and dark conditions until use.

### 2.3. Microarray Incubation, Scanning, and Image Processing

Sample incubation on the antibody microarrays was performed on a HS4800 hybridization station (Tecan, Männedorf, Switzerland). First, slides were allowed to settle at room temperature for about 30 min before being washed with phosphate buffered saline (PBST: 137 mM NaCl, 12 mM phosphate, 2.7 mM KCl, pH 7.4 with 0.1% Tween-20). After blocking with a casein-based blocking solution (Candor Biosciences, Weißensberg, Germany) for 1 h at room temperature, 5 μg of each labeled protein sample was taken up in blocking buffer containing 1% Triton and 1x protease inhibitor (Roche). Tumour samples were mixed with the extracts isolated from the respective adjacent non-tumorous tissue but labeled with a different fluorescent dye. Incubation on the microarrays was for 8 h at 4 °C. Then, the microarrays were washed with PBST, rinsed with water and dried.

### 2.4. Data Analysis and Sample Classification

Scanning of the slides was performed with a PowerScanner (Tecan) at a resolution of 10 μm, maintaining laser power and the photomultiplier constant. Relevant parameters, such as the concordance of the two colour detection channels, were carefully validated throughout. Spot segmentation was performed with GenePix Pro 6.0 software package (Molecular Devices, Sunnyvale, CA, USA). The complete data set is made available as Supplemental Table 1. The spot signals were analysed with R-Bioconductor using a one-factorial linear model fitted with LIMMA package, which resulted in a *t*-test based on moderated statistics. Adjusted *p*-values were calculated [20]; protein variations with *p*-values less than 0.05 were considered significant. There was not cut-off on the basis of the degree of variation; the only measure was significance. After sample normalization, an unsupervised hierarchical clustering was performed to check for technical and handling artifacts in the data. In order to show how the single-channel, log_2_ expression signals of the arrays can be used for discriminating between tumor and non-tumorous tissue, a Random Forest classifier [20] was trained using tissue type as response variable and the log_2_ protein expression signals of IGFBP7, S100A9, IL-10 and MUC6 as explanatory variables. This model was validated with 500 bootstrap iterations, resulting in (out-of-bag) a receiver operating characteristic curve and a corresponding area under the curve (AUC) value. For training and validating the classifier, the R-packages randomForest [21] and caret [22] were applied, using the default settings for classification.

### 2.5. Immunohistochemistry

Immunohistochemistry analysis was performed on paraffin sections of five tissue samples. Deparaffinization and rehydration was performed on the sections via Roticlear and a diluted series of alcohol. Thereafter, the samples were boiled twice in 10 mM citrate buffer for 10 min. The slides were incubated at 4 °C overnight with a 1:100 dilution of primary antibodies IGFBP7 (ab171085, Abcam, Cambridge, UK) or S100A9 (ab24111, Abcam), respectively. Then, biotinylated secondary horse anti-mouse antibody (diluted 1:200; Vector, BA-2000) was added and incubated for 45 min. For chromogen staining, streptavidin-HRP conjugate (1:100; Perkin Elmer, NEL700001KT) was added followed by biotinyl-tyramide. Antibody detection was performed by staining with 3,3′-diaminobenzidine (DAB) and counterstaining with Haemalaun. After that, sections were rehydrated, fixed in ethanol/xylol and examined microscopically.

## 3. Results

Sixteen tumor tissue samples (Table 1) were surgically removed along with adjacent normal tissue from patients diagnosed with gastric adenocarcinoma. Total protein was extracted and analyzed on high content microarrays that consisted of 813 antibodies directed against 724 cancer-related proteins [15]. The sample pairs were labeled with two different fluorescent dyes and incubated together on the microarrays. After incubation and image acquisition, the data was normalized in order to account for differences in labeling efficiencies (Supplemental Table S1). The resulting signal intensities were subjected to an unsupervised clustering for the identification of any factor that may introduce a bias. The samples were found clustered according to the tissue type, suggesting that the clustering and segregation of normal and tumor samples were based on the differential abundance of proteins. No other factor, such as patient gender or patient age, date of surgery, order of protein preparation, labeling, array incubation or scanning, could be identified that may have influenced the clustering.

**Figure 1:**
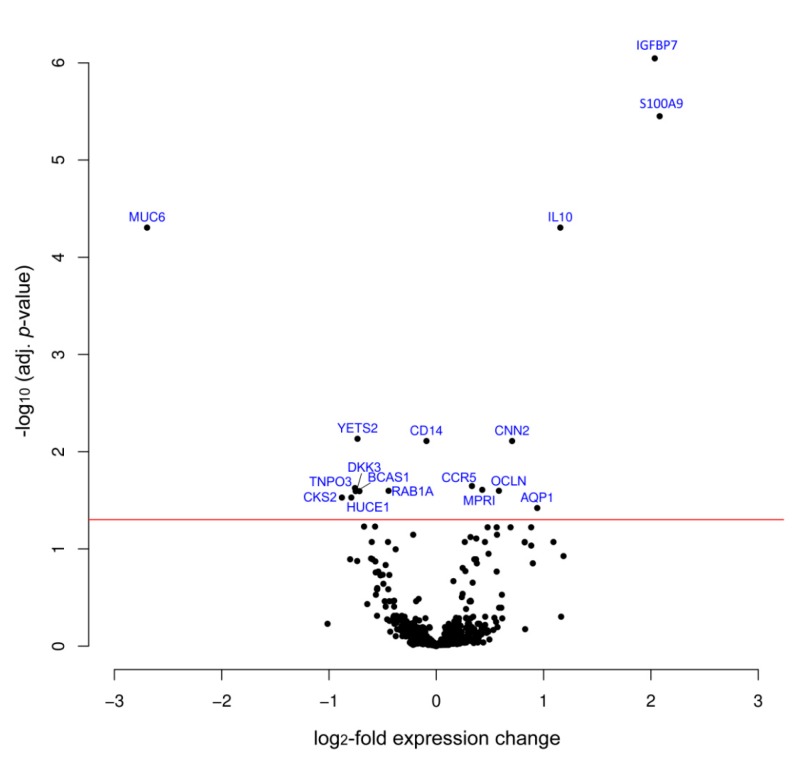
Volcano plot of protein expression variations. Protein expression in tumor and adjacent non-tumorous tissues was compared by means of the antibody microarray. The degree of variation and its significance are shown; the horizontal red line indicates an adjusted *p*-value of 0.05. The black dots represent the proteins analyzed. While most of them fall below this threshold, 17 proteins exhibited significant differences. Some protein names are given; the complete list and the relevant data are shown in Table 2.

Of the 724 proteins against which there were antibodies on the array, only 17 proteins exhibited significantly differential abundance in the normal and tumor patient samples (Figure 1; Table 2). This number is surprisingly small, given the fact that the antibodies target mostly proteins, which are tumor-associated. In other analyses using the same platform, but comparing tumor versus healthy tissue (rather than non-tumorous material from next to the tumor), more than half of the proteins identified by the microarray antibodies always exhibited significant variation (e.g., [21,22]). One reason for the very few changes observed in gastric cancer could be its high degree of heterogeneity. While some proteins are differentially expressed in some tumors, only few proteins are similarly different in abundance in most or all samples. Another explanation could be that tissue that is located adjacent to the tumor is not that different molecularly to the actual tumor tissue, although no tumor cells are present.

**Figure 2:**
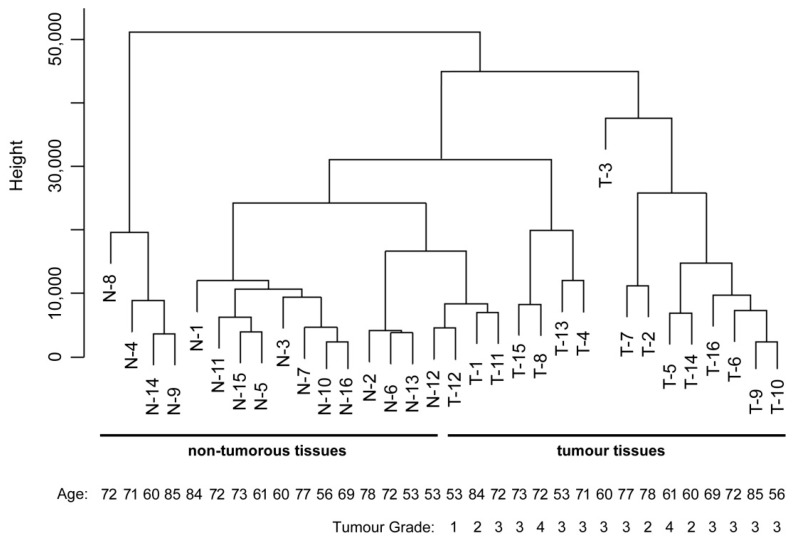
Hierarchical clustering of the samples. Based on the expression of IGFBP7, S100A9, IL-10 and MUC6, the samples were subjected to hierarchical clustering. In most cases, there was a clear separation of non-tumorous and tumor samples. Below, for each sample, the age of the patients, from whom the tissues were isolated as well as the tumor grades are shown.

Within the group of 17 proteins, four exhibited the most pronounced expression difference: IGFBP7, S100A9 and IL-10 were found strongly up-regulated in tumor, while MUC6 was markedly less abundant compared to the reference tissue. The power of discriminating tumor and control samples was determined for the markers individually or in combination. The complete data set obtained from the microarray analysis yielded discrimination worse than that of the four proteins with the strongest variation alone. Their combined signature was found to perform best. A clustering based on the four markers led to a separation of the non-tumorous and tumor tissues (Figure 2). While yielding a good discrimination of tumor and non-tumorous samples, separation was not perfect. A particular example was the sample pair isolated from patient No. 12: both specimens behaved nearly identically as indicated by their clustering. They were the only sample pair isolated from a patient with a tumor grading of 1. However, two other tumors—T-1 and T-11—were also not overly different from the average control tissue, although clearly distinct from their matched non-tumorous control sample. A principle component analysis produced a very similar picture. The addition of other marker molecules improved the separation of individual sample pairs. None of them was applicable to several pairs, however, and all of them decreased the overall performance.

**Figure 3:**
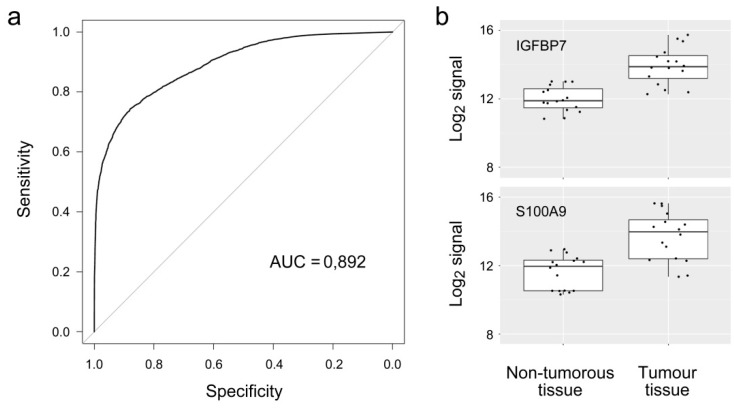
Accuracy of diagnosis. (**a**) The expression signature consisting of the expression of IGFBP7, S100A9, IL-10 and MUC6 was used for performing a Receiver Operating Characteristic curve analysis. The resulting area under the curve (AUC) was 89.2%; (**b**) Box plots are shown for IGFBP7 and S100A9. The black dots stand for the individual measurements. The median as well as the upper and lower quartile are represented by the boxes.

**Figure 4:**
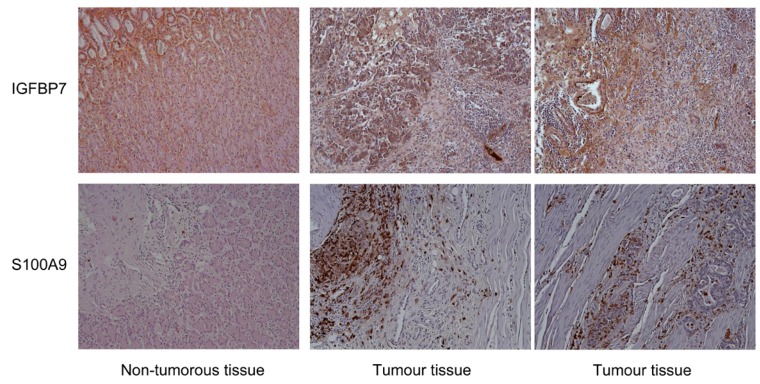
Typical results of immunohistochemical analyses. Marker molecules identified by the microarray analysis were validated and confirmed by immunohistochemistry (magnification ×100) on tissue slices made from an independent set of tumor tissues and stomach samples from donors who had no cancer. Dark brown color is indicative of the presence of the respective protein.

Apart from a variation between individual tumors, the distance of the non-tumorous tissues from the actual tumor location in the patient did differ. Unfortunately, no annotated information is available about this. Variation in the difference between tumors and controls could therefore be brought about by both biological as well as experimental heterogeneity. The accuracy of discriminating between tumor and adjacent non-tumorous tissue was evaluated more quantitatively by a Receiver Operating Characteristic curve analysis. An AUC value of 89.2% was calculated (Figure 3) with the four marker molecules. For confirmation, immunohistochemistry analyses were performed on samples representing tumor or tissue obtained from healthy individuals. In all cases, the results were in agreement with the microarray data (e.g., Figure 4).

## 4. Discussion

We focused our study on a comparison of gastric tumors and tissues close to them, rather than comparing stomach tissues from healthy donors and patients with gastric adenocarcinoma. Thereby, we aimed at avoiding any markers for effects such as inflammation, which are related to carcinogenesis but are not necessarily specific for cancer. While the number of samples was relatively small, the study design was such that identified markers had to be rather selective for tumor. The analysis yielded only relatively few proteins that exhibited significant differences in expression. While this was partly expected, the very low number came as a surprise. One reason could be a kind of cancer field defect, which is defined as a biological cancerization process in which the cells of relatively large areas beyond the actual tumor are exhibiting changes similar to the ones of the actual tumor [23,24,25]. As opposed to intercellular communication in the microenvironment, any molecules responsible for such a field defect had to be transported over relatively longer distances, since the non-tumorous tissue samples studied here had a distance to the tumor of up to one centimeter. Heterogeneity could be another explanation for the small number of differentially expressed proteins. While it should be less pronounced at the protein level, there could nevertheless be different protein combinations that result in a particular cancer phenotype. The number of common proteins would then be small. However, particularly these molecules may be essential for the tumor and may consequently be specific markers for diagnosis as well as proteins that are prime target candidates for therapy.

Among the few significantly different proteins were four molecules—IGFBP7, S100A9, IL-10 and MUC6—that were set apart from the others by their degree of variation, which was both particularly strong and significant. In combination, they were able to differentiate tumor tissue from adjacent non-tumorous tissue with good accuracy. The immunohistochemical analysis indicates that the discriminative power may be even better when comparing healthy and tumor tissues. However, the number of samples analyzed was too small to validate this assumption sufficiently. Currently, we are in the process of studying this on a much larger number of patient and control samples. In this early-stage trial, we also aim at investigating the prognostic power of the biomarkers.

IGFBP7 was the molecule that exhibited the most significant variation. There have been conflicting reports about the correlation of its expression and tumor progression and survival. A recent study showed that an elevated IGFBP7 expression level in gastric cancer is associated with invasion, tumor progression and recurrence as well as poor survival [26]. As opposed to this, decreased IGFBP7 expression was also reported to be associated with tumor progression and poor survival in gastric cancer [27]. These controversial results could be explained by the fact that IGFBP7 is not homogeneously expressed [28] and that IGFBP7 signaling in stromal fibroblasts could override the tumor-suppressor function on epithelial cells.

S100A9 is a member of the S100 family and is overexpressed in various forms of cancer. It was reported to be up to five times more abundant in the plasma of patients with colorectal cancer, revealing its high diagnostic potential [29]. Immunohistochemistry analyses of gastric cancer samples showed that S100A9 was exclusively located in inflammatory cells, such as macrophages and neutrophils, infiltrating primary tumor tissues while all gastric cancer cells or cells adjacent to gastric mucosa did not express S100A9 [30]. Surprisingly, patients with a high S100A9 cell count had a favorable prognosis. S100A9 induces phosphorylation of MAPK and promotes the activation of NF-κB [31]. Several studies have therefore examined a possible role in tumor cell migration and invasion [32,33]. The molecular mechanisms of S100A9 in gastric cancer invasion and migration are not fully understood, however.

IL-10 is a cytokine with immunosuppressive properties which is frequently elevated in the tumor microenvironment and correlates with a bad outcome [34]. It has been reported to be elevated in the blood of gastric cancer patients [35]. IL-10 represses the expression of inflammatory cytokines, such as TNF-α, Interleukin-6 and Interleukin-1-β, in macrophages. This role of IL-10 fostered the assumption that it undermines the immune response to cancer [36]. Upregulation of IL-10 has been suggested to be responsible for the persistence of inflammation and might facilitate tumor progression [37].

MUC6 is a highly glycosylated, secreted mucin that is involved in forming a physical barrier, which is critical for the protection of the epithelium. Its low expression was reported to contribute to the malignant transformation of gastric epithelial cells [38]. In general, mucins function by limiting the inflammatory responses at the interface with the environment. Deregulation of mucin production has therefore provided an important link between inflammation and cancer [39].

## 5. Conclusions

Overall, the particular set-up of our analysis—a comparison of tumors to non-tumorous tissues that had been directly adjacent to the tumors—identified a few markers which had been implicated in the carcinogenesis of gastric tumors before. More importantly, however, it ruled out a lot of other potential markers, in particular candidates that had been suggested on the basis of mRNA or protein profiling analyses comparing tissues from patients and healthy donors [40], which may not be tumor-specific, however. In addition, because of the pairwise evaluation of samples isolated from the same patient, the influence of variations between the individual donors could be ruled out. Instead, the analysis focused exclusively on the differences between tumor and non-tumorous tissues. Toward an application for diagnostic purposes, further studies need to be performed. In particular, abundance variations of these specific markers in plasma or serum of cancer patients would be of high utility. It is more likely that markers, which exhibit a strong expression increase, will be useful to such ends.

## Figures and Tables

**Table 1:** Characteristics of the tumor samples.

Sample Number	Patient Gender	Patient Age	Location in Stomach	Tumour Grade	Lauren Classification	Number of Positive Lymph Nodes
1	Male	84	Corpus	2	Intestinal	1
2	Male	78	Cardia	2	Intestinal	0
3	Male	60	Cardia	3	Intestinal	7
4	Male	71	Cardia	3	Diffuse	5
5	Male	61	Antrum	4	Mixed	8
6	Female	72	Antrum	3	Intestinal	2
7	Female	77	Antrum	3	Intestinal	0
8	Male	72	Antrum	4	Mixed	0
9	Male	85	Antrum	3	Mixed	0
10	Male	56	Corpus	3	Diffuse	1
11	Male	72	Cardia	3	Intestinal	21
12	Male	53	Corpus	1	Intestinal	0
13	Male	53	Spread	3	Intestinal	12
14	Male	60	Spread	2	Intestinal	10
15	Male	73	Corpus	3	Intestinal	12
16	Female	69	Spread	3	Intestinal	16

**Table 2:** Proteins that exhibited significant abundance variations between tumor and adjacent non-tumorous tissue.

Protein	Variation log_2_FC	Adj. *p*-Value	Swissprot No.	Full Protein Name
Proteins with most significant and strongest variation in expression				
IGFBP7	2.04	8.99 × 10^−7^	Q16270	Insulin-like growth factor-binding protein 7
S100A9	2.08	3.54 × 10^−6^	P06702	S100 calcium binding protein A9
IL10	1.16	4.96 × 10^−5^	P22301	Interleukin 10
MUC6	−2.70	4.96 × 10^−5^	Q6W4X9	Mucin 6
Other proteins of higher abundance in tumor				
CNN2	0.71	7.77 × 10^−3^	Q99439	Calponin-2
CCR5	0.33	2.26 × 10^−2^	P51681	C-C chemokine receptor type 5
MPRI	0.43	2.41 × 10^−4^	P11717	Cation-independent mannose-6-phosphate receptor
OCLN	0.58	2.53 × 10^−2^	Q16625	Occludin
AQP1	0.94	3.80 × 10^−2^	P29972	Aquaporin 1
Other proteins of lower abundance in tumor				
YETS2	−0.73	7.37 × 10^−3^	Q9ULM3	YEATS domain-containing protein 2
CD14	-0.09	1.07 × 10^−2^	P08571	Monocyte differentiation antigen CD14
TNPO3	−0.75	2.35 × 10^−2^	Q9Y5L0	Transportin-3
BCAS1	−0.72	2.53 × 10^−2^	O75363	Breast carcinoma-amplified sequence 1
RAB1A	−0.44	2.53 × 10^−2^	P62820	Ras-related protein Rab-1A
DKK3	−0.74	2.53 × 10^−2^	Q9UBP4	Dickkopf-related protein 3
CKS2	−0.88	2.97× 10^−2^	P33552	Cyclin-dependent kinases regulatory subunit 2
HUCE1	-0.79	2.97× 10^−2^	O43159	Cerebral protein 1

FC: Fold Change.

## Supplementary Materials

The supplementary material is available online at http://www.mdpi.com/2076-3905/5/3/19/s1.
